# An in vitro methodology for experimental simulation on the natural hip joint

**DOI:** 10.1371/journal.pone.0272264

**Published:** 2022-08-18

**Authors:** David Jimenez-Cruz, Mudit Dubey, Tim Board, Sophie Williams

**Affiliations:** 1 Institute of Medical and Biological Engineering, School of Mechanical Engineering, University of Leeds, Leeds, United Kingdom; 2 Wrightington Wigan and Leigh NHS Trust, Wigan, United Kingdom; University of Life Sciences in Lublin, POLAND

## Abstract

Different hip pathologies can cause geometric variation of the acetabulum and femoral head. These variations have been considered as an underlying mechanism that affects the tribology of the natural hip joint and changes the stress distribution on the articular surface, potentially leading to joint degradation. To improve understanding of the damage mechanisms and abnormal mechanics of the hip joint, a reliable in-vitro methodology that represents the in vivo mechanical environment is needed where the position of the joint, the congruency of the bones and the loading and motion conditions are clinically relevant and can be modified in a controlled environment. An in vitro simulation methodology was developed and used to assess the effect of loading on a natural hip joint. Porcine hips were dissected and mounted in a single station hip simulator and tested under different loading scenarios. The loading and motion cycle consisted of a simplified gait cycle and three peak axial loading conditions were assessed (Normal, Overload and Overload Plus). Joints were lubricated with Ringer’s solution and tests were conducted for 4 hours. Photographs were taken and compared to characterise cartilage surface and labral tissue pre, during and post simulation. The results showed no evidence of damage to samples tested under normal loading conditions, whereas the samples tested under overload and overload plus conditions exhibited different severities of tears and detachment of the labrum at the antero-superior region. The location and severity of damage was consistent for samples tested under the same conditions; supporting the use of this methodology to investigate further effects of altered loading and motion on natural tissue.

## Introduction

Hip osteoarthritis causes debilitating pain and loss of function and affects over 300 million people worldwide [1]. The causes of hip osteoarthritis are multifactorial; however, its onset and progression can be accelerated by abnormal biomechanics of the joint [2, 3]. The mechanical environment of the natural hip joint is related to hip geometry, this is altered in conditions such as femoroacetabular impingement, developmental dysplasia or slipped capital femoral epiphysis [3].

Despite evidence from clinical and in silico studies [4, 5] demonstrating that the mechanical environment contributes to degradation in the hip, in vitro studies assessing this are limited [6–8], particularly in terms of considering the effects of repeated loading cycles. Groves et al. [9] have reported on an experimental simulation of the natural hip where the head and acetabular were inverted and subjected to a simplified loading and motion in one direction, running at a frequency of 1 Hz for 7,200 cycles (2 hours). Other studies have applied more realistic load and motion conditions (Ng et al. [10]) and assessed the contributions of the capsule and ligament to the mechanical behaviour of the joint using a robot arm. However, these studies did not assess the tribology of the joint over time.

While the in vitro assessment of the mechanical environment on the natural hip is limited, total hip replacements have been widely studied using experimental hip simulators. These in vitro simulators are able to apply clinically relevant loading and motion cycles to hip replacements in a lubricant that mimics synovial fluid. The effects of parameters such as the material the hip replacement is made from, its diameter, the relative position of the components; as well as load and motion cycles applied have been assessed [6–8].

In order to develop further experimental simulation of the natural hip, a reliable in-vitro methodology where the position of the sample, the congruency of the bones and the demand of load and motion are clinically relevant is needed. Such a methodology can develop our understanding of abnormal mechanics of the joint and possible damage mechanisms. The aim of this study was to develop and validate a methodology for experimental simulations of the natural hip joints using an electro-mechanical simulator and use the developed methodology to demonstrate the effects of altering the mechanical environment (increased loading). We hypothesised that the developed method would enable us to differentiate between loading environments thought observation of damage to the natural hip cartilage and labrum.

## Materials and methods

Porcine hip joints were selected for use because of their availability and lack of variation compared to cadaveric human tissue. The materials section describes the preparation of the porcine samples for testing; and the methods section describes the experimental simulator that was used, the loading and motion protocols adopted for testing and the post-test analysis.

### Materials: Preparation of porcine hips

Twelve right hind legs from 6-month-old pigs were obtained within 24-48hrs of slaughter. Animals were slaughtered at the local abattoir (John Penny & Son’s, Leeds, UK) and taken from the food chain.

**Fig 1:**
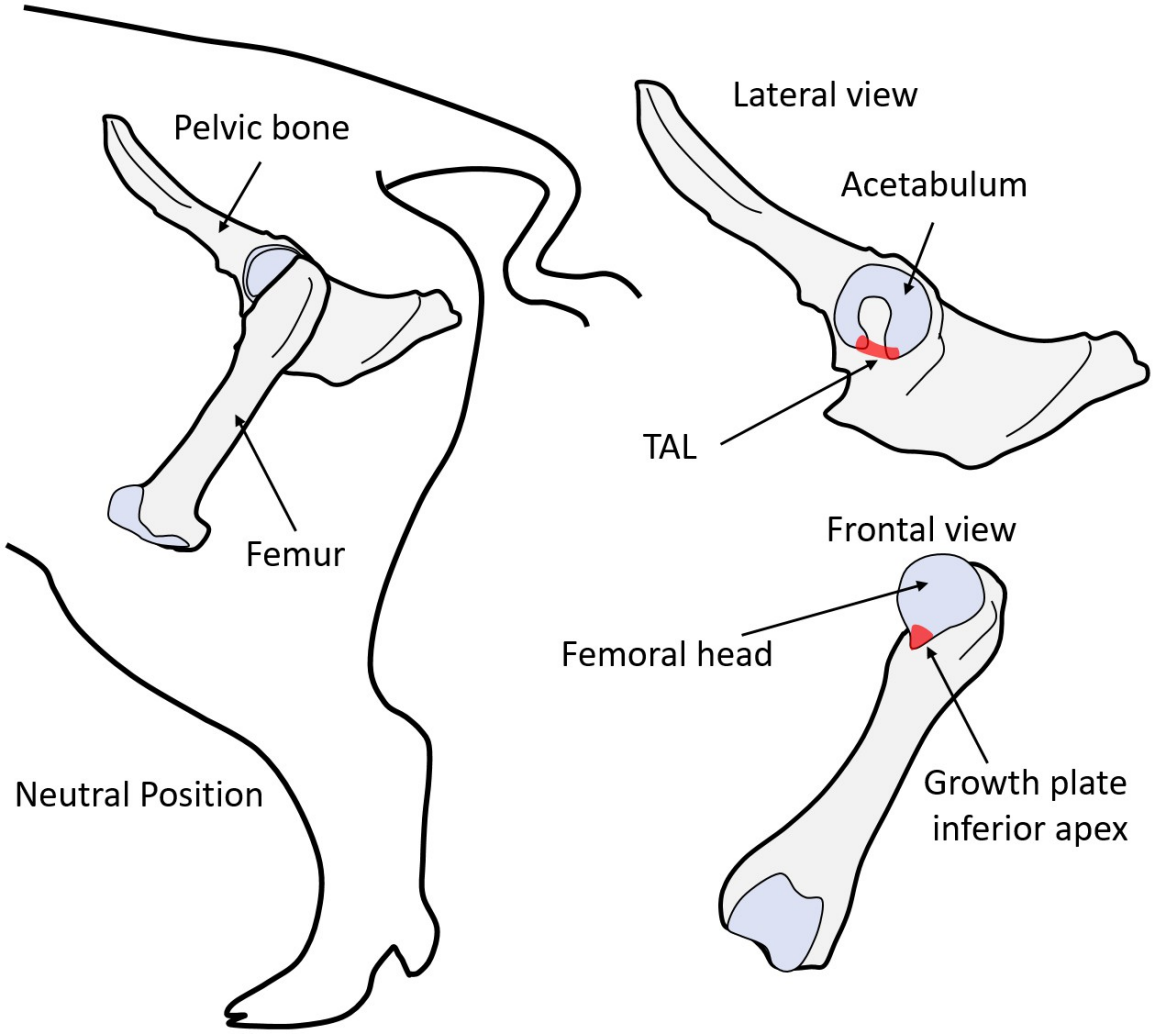
Neutral position of the porcine hip joint in vivo. During dissection, the landmarks (red) when viewed from the inferior direction were aligned. Landmarks were used to maintain the alignment of the bones during cementing and mounting. TAL is the transverse acetabular ligament.

**Fig 2:**
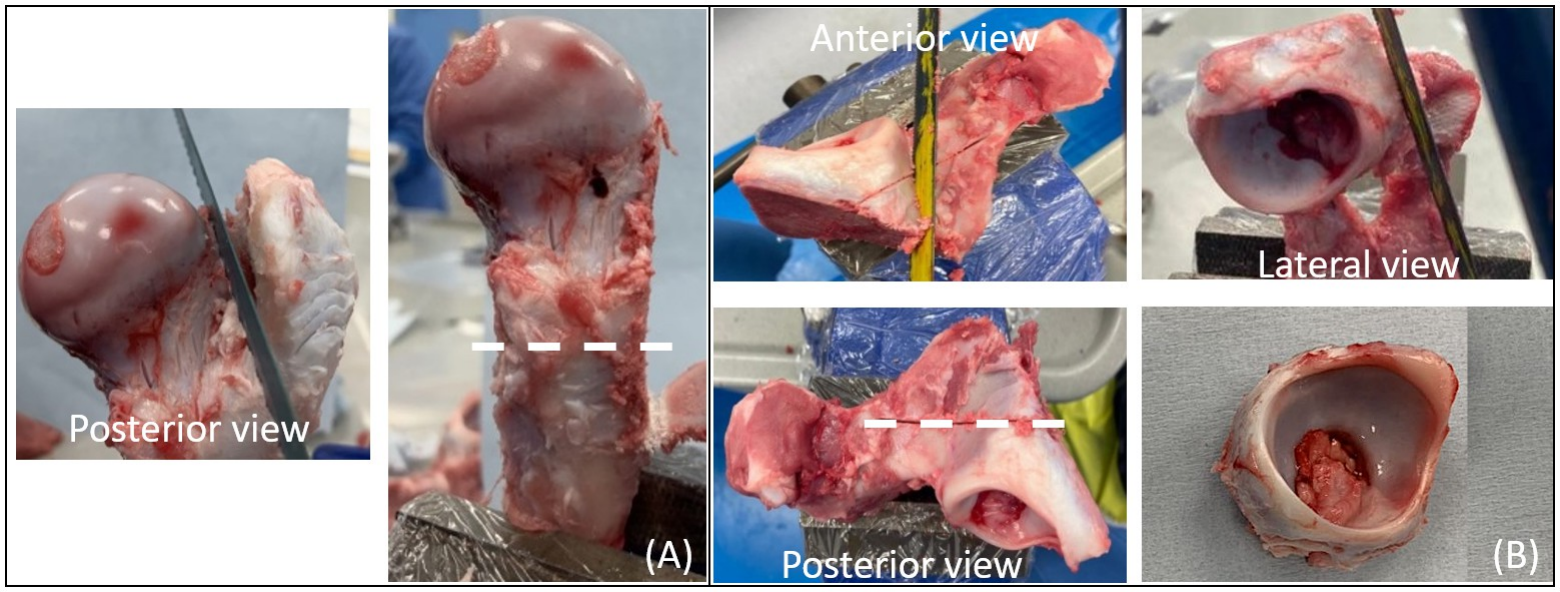
Preparation of porcine hip joint. (A) Removal of the grater trochanter and cut on the femoral diaphysis. Posterior view of the proximal femur indicating the resection of the greater trochanter and the positon for the transversal cut on the femoral shaft. (B) Resection of the pelvic bones. Lateral, anterior and posterior views indicating the three periacetabular cuts executed at approximately 15 mm from the acetabular rim.

All soft tissue surrounding the hip joint was removed by dissection. Prior to disarticulating the joint (by removal of the capsule and ligamentum teres), the neutral position between the femur and acetabulum was assumed to be when the centre of the transverse acetabular ligament (TAL) aligned with the inferior apex of the growth plate in the head in the direction of the lesser trochanter. At this position, the capsule also exhibited a neutral visible tension, indicating the resting position of the joint (Fig 1). The residual tissue from the capsule was then removed and ligament teres divided, avoiding damage to the articular surfaces and labrum. Throughout sample preparation, cementing and mounting, cartilage surface hydration was maintained by frequent spraying with PBS. The femur was resected approximately 65 mm distal to the femoral head centre and the greater trochanter was removed so as to continue the curvature of the femoral head to the femoral diaphysis (Fig 2(A)). Resection of the greater trochanter was necessary to allow the desired range of motion (RoM) to be reached during simulation without causing impingement with the acetabulum or the simulator fixtures (porcine hips have a more prominent greater trochanter compared to humans).

On the pelvic side, bone was resected so approximately 5 mm of peri-acetabular bone around the acetabulum was maintained and the laburm remained intact. The posterior periacetabular bone was resected parallel to the acetabular rim in order to avoid any interference with the acetabular fossae soft tissue that could have led to cement flowing into the articular surface (Fig 2(B)).

**Fig 3:**
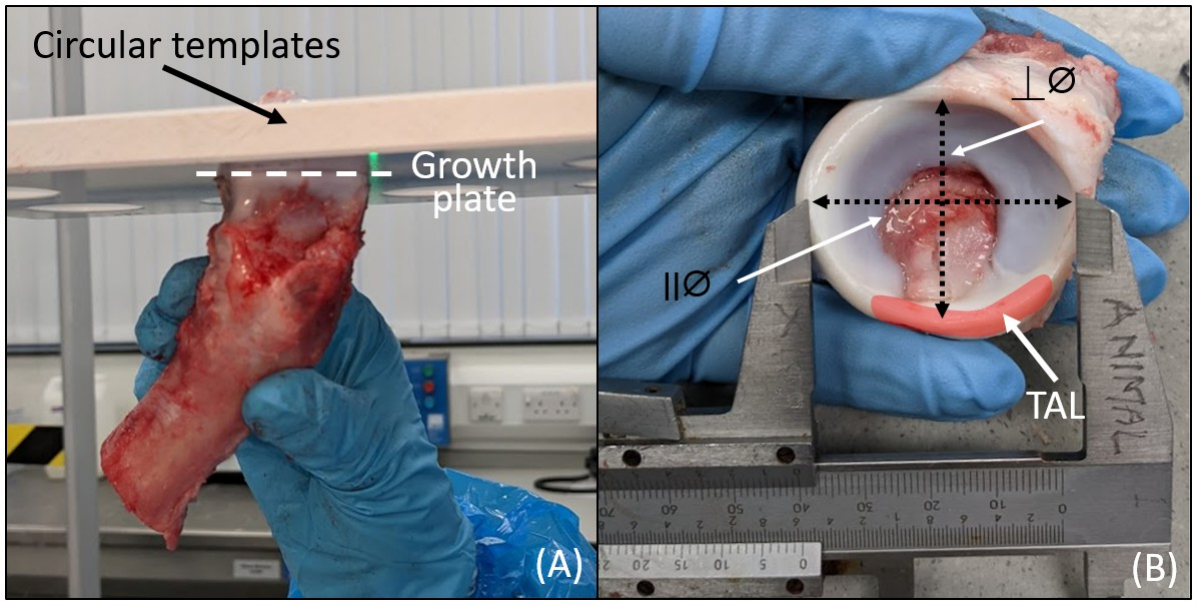
Preparation of porcine samples for experimental simulation. (A) Measurement of femoral head diameter with the circular templates, diameter was recorded at parallel to the epiphyseal line (growth plate) whereby at this point the head easily fitted through a template of defined size. (B) Measurement of acetabular diameters taken parallel to the TAL (ll∅); and perpendicular to TAL (⊥∅).

The diameters of the head and acetabulum were recorded. Head diameters were measured using circular templates at the centre of the head in a parallel plane to the epiphyseal line (Fig 3(A)). Positioning the TAL inferiorly, acetabular diameters were measured with a calliper, taking the measurement at the interior edge of the labarum parallel and perpendicular to the TAL (Fig 3(B)). Sample preparation took approximately 1 hour from the point of receipt it from the abattoir to being prepared to commence cementing.

**Fig 4:**
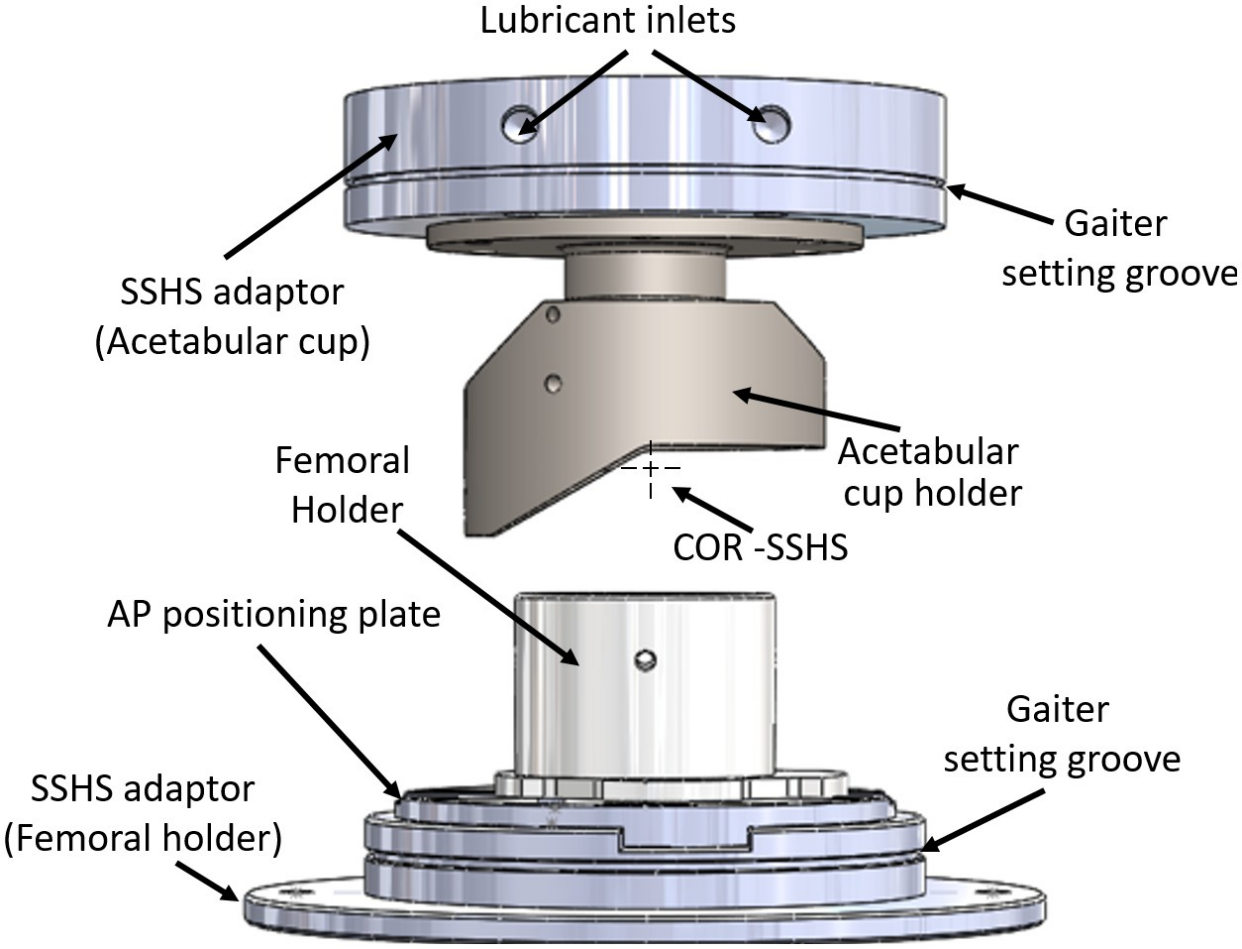
Overview of fixtures used for the porcine head and acetabulae for experimental simulation on the single station hip simulator (SSHS). Centre of rotation (COR) of the simulator is indicated.

The method of sample mounting was developed to ensure three main objectives: 1) the COR of the hip was coincident with the COR of the simulator, 2) the neutral position of the joint and the relative position between bones were anatomically correct, and 3) there was no impingement of the fixtures within the RoM used in the experimental testing. An overview of the fixtures used are shown in Fig 4. The femoral head and acetabulae samples were mounted using PMMA (poly(methyl methacrylate)) bone cement in bespoke fixtures to ensure the centres of rotation were coincident with the centre of rotation (COR) of the experimental simulator. Curing time for the cement was approximately 20 minutes at room temperature (21° C) per component, the acetabulum was cemented first and then used to position the femoral head, hence the full process took an average of 40 min per hip.

**Fig 5:**
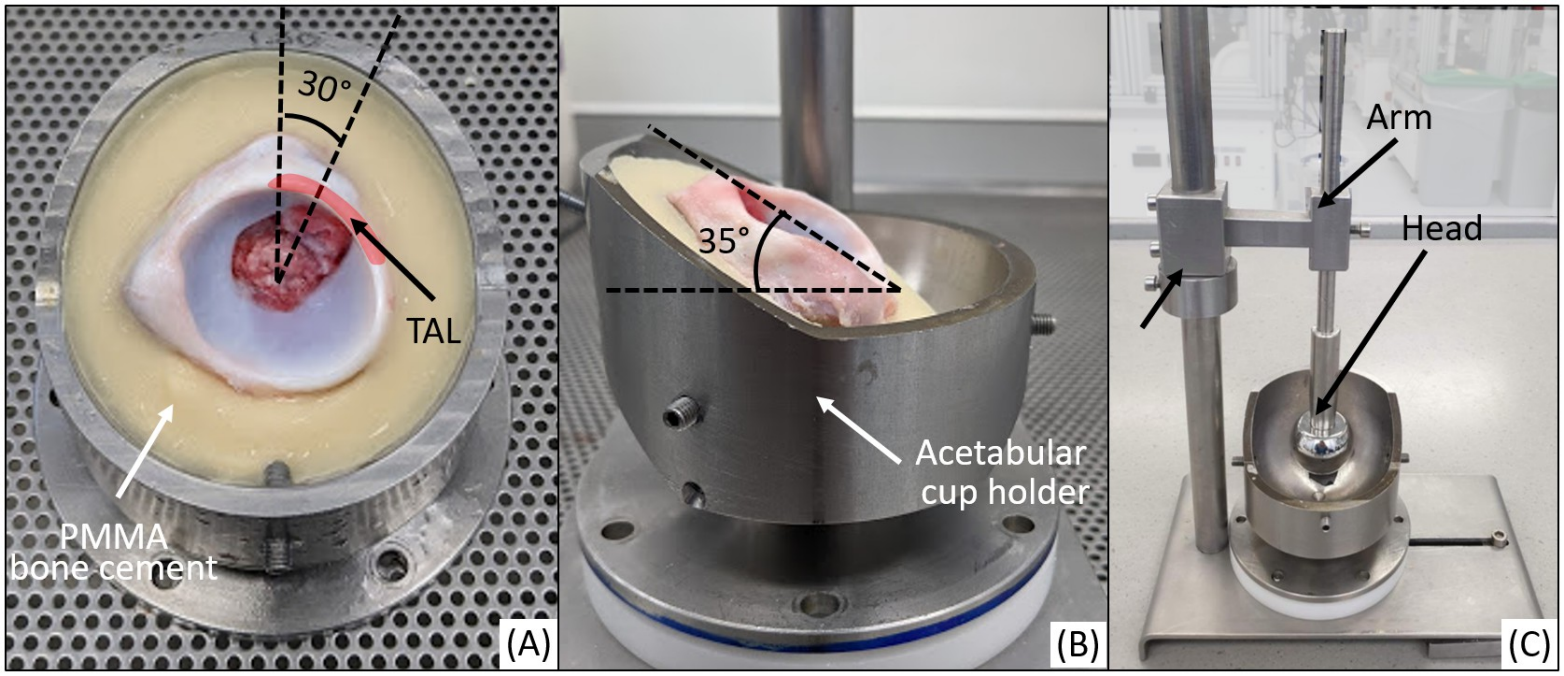
Experimental acetabular cup holder. Inverted compared to position in Fig 4 and including a cemented porcine acetabulum showing: (A) the acetabular version angle (whereby TAL indicates the transverse acetabular ligament), (B) the acetabular inclination angle (C) acetabular height set using a metal head attached the fixture via an arm.

In order to mount the acetabulae in an anatomically correct position, the acetabulum was placed with the TAL positioned towards the raised portion of the acetabular cup holder (Fig 5(A)). The acetabular version was set at a 30° angle between the sagittal plane and a plane through the centre of the TAL. The inclination angle of the acetabulum was 35° between the acetabular rim plane and the transverse plane (Fig 5(B)). A movable collar-arm-head assembly was used to centre and to set the height of the acetabulum (Fig 5(C)).

**Fig 6:**
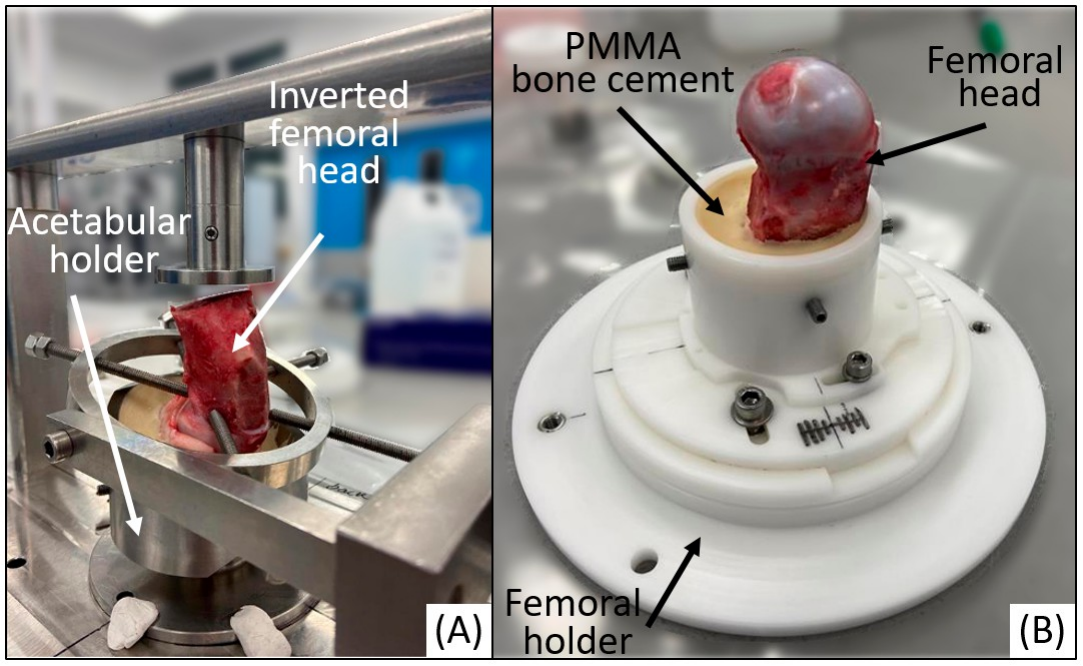
Experimental set up of the femoral head. (A) Femoral head position fixed in an inverted position so it is anatomically aligned with the acetabulum using a custom potting fixture; (B) Femoral head cemented into the femoral holder (as shown in Fig 4) simulator fixture.

The (inverted) femur was placed in anatomical alignment in the acetabulum and fixed using a custom potting fixture (Fig 6(A)). The sample was then rotated and fixed into a pot with bone cement (Fig 6(B)). Following set up and prior to testing, samples and fixtures were submerged in Ringer’s solution in an oscillating bath for 10 minutes to remove any cement or bone debris.

### Methods: Experimental simulator testing

**Fig 7:**
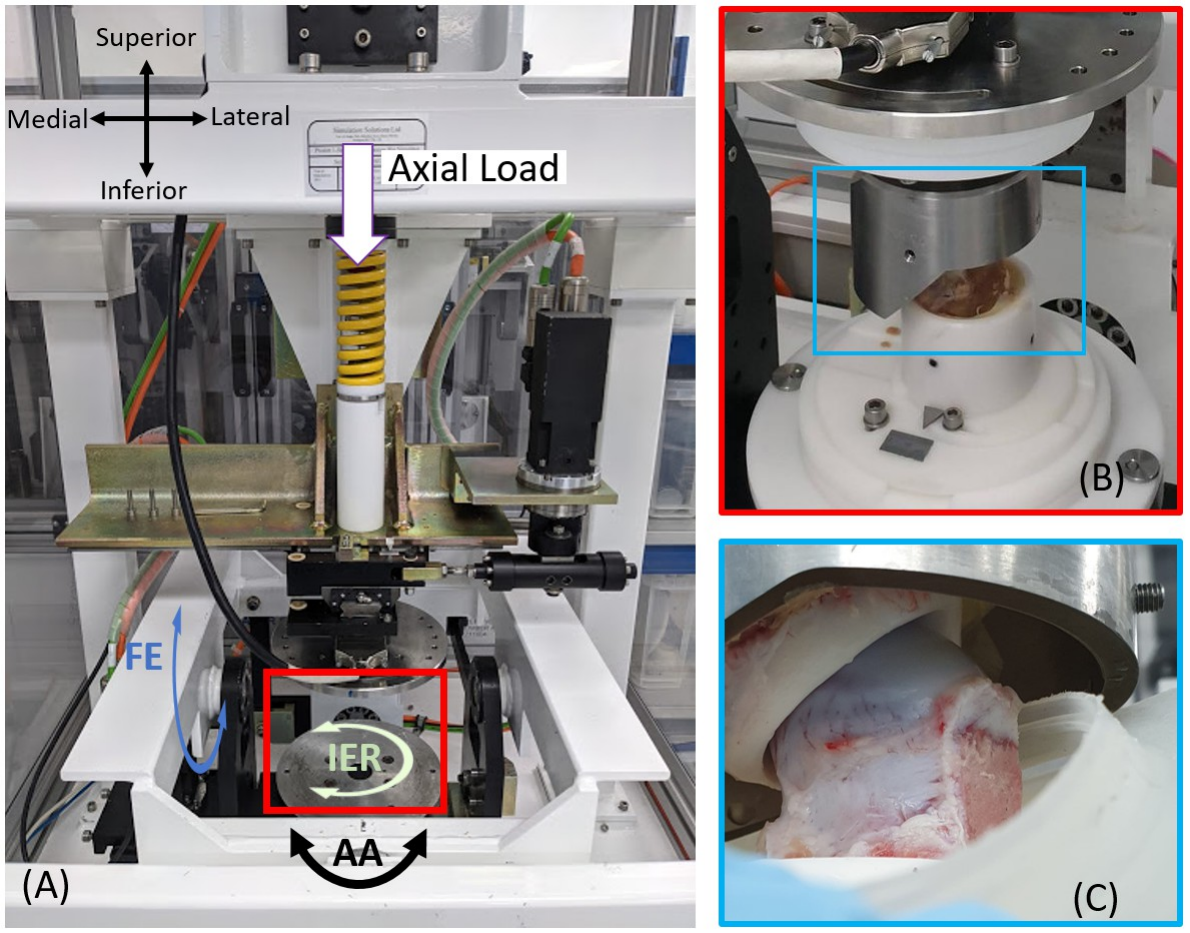
Simulator and mounted sample. (A) Single station hip simulator (SSHS); (B) right porcine hip sample cemented and mounted for simulation; (C) Femoral head and acetabulum position prior simulation. FE: flexion-extension, AA: abduction-adduction; IER: interior-exterior rotation.

An electro-mechanical Single Station Hip Simulator (SSHS); Simsol Simulation Solutions Ltd., Stockport, UK, was used to perform the in-vitro experimental simulations (Fig 7). The SSHS is a multi-axis simulator that can apply flexion-extension (FE), abduction-adduction (AA) and interior-exterior (IE) motions to the femoral head and a compressive axial force up to 4500 N to the acetabulum.

The porcine hip joints, mounted in holders as previously described (Figs 5 and 6), were positioned in the simulator in an anatomically correct set up (Fig 7). To ensure that the articular surfaces were covered by the fluid throughout the test, a silicon gaiter encapsulated the samples and was filled with Ringer’s solution (1000 ml) for lubrication [11].

### Test conditions

Four porcine hips were tested under conditions representative of normal peak load (NOR). The load and motion profile selected was based on the ISO standard for total hip replacement (THR) wear testing (ISO 14242). The axial load was scaled due to the reduced load expected in a quadruped and the average mass of the animals [12–15]. The peak load was 900 N (compared to 3 kN in the ISO standard used in human THR testing) [15].

**Fig 8:**
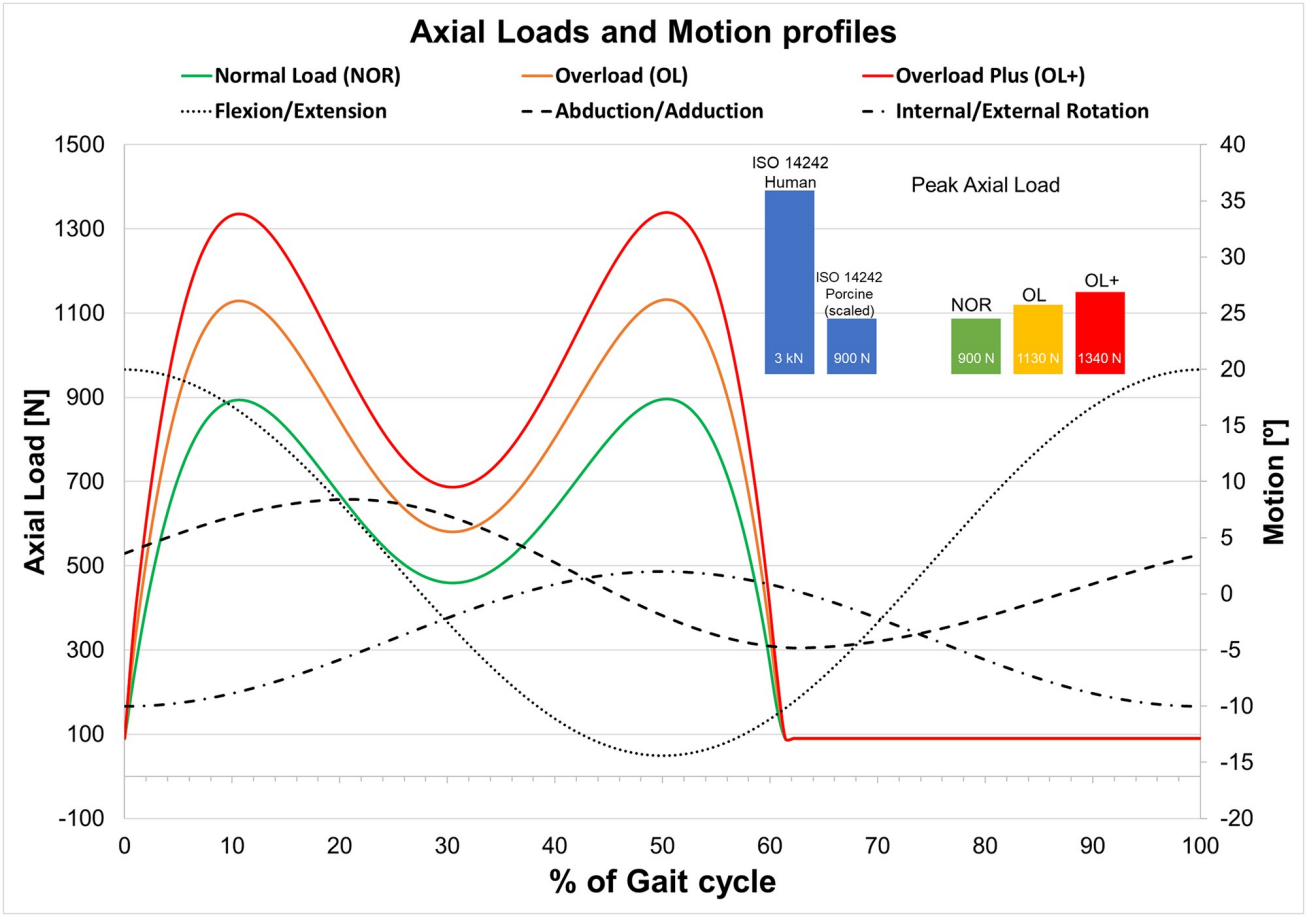
Loading profiles and motion for the experimental simulations. Coloured lines show the axial loading input for the different scenarios (Green = normal load NOR; amber = overload OL and red = overload plus OL+). Black dotted lines show the motion profiles followed during all the simulations. Inset bar charts show the relative peak load in ISO 14242 axial loading and the scaled loading used in this study.

The effects of mechanical loading on the porcine hip was considered in further testing with increased loading, as follows: the overload condition (OL, n = 4) used the load profile described above with an increase in peak load to 1,130 N corresponding to an approximate increase of 25% of the NOR loading and the overload plus (OL+, n = 4) condition with peak load of 1340 N which represented an approximate increase of 50%. In all loading profiles (Fig 8) the load was applied through the acetabulum and the swing phase remained constant (90N).

The experimental simulator applied simultaneous motions representative of human gait to the femoral head, as follows: ± 20 Flexion-Extension (FE), 8.8 to -4.8 degrees of Abduction-Adduction (AA) and 2 to -10 degrees of Internal-External Rotation (IER), (Fig 8). The medial lateral axis was free, so the tissue followed the natural motion and any additional torque produced by the restriction of the carriage movement was avoided. All the tests were conducted at 1Hz for a total 14,400 cycles (4 hrs).

### Post test analysis

**Fig 9:**
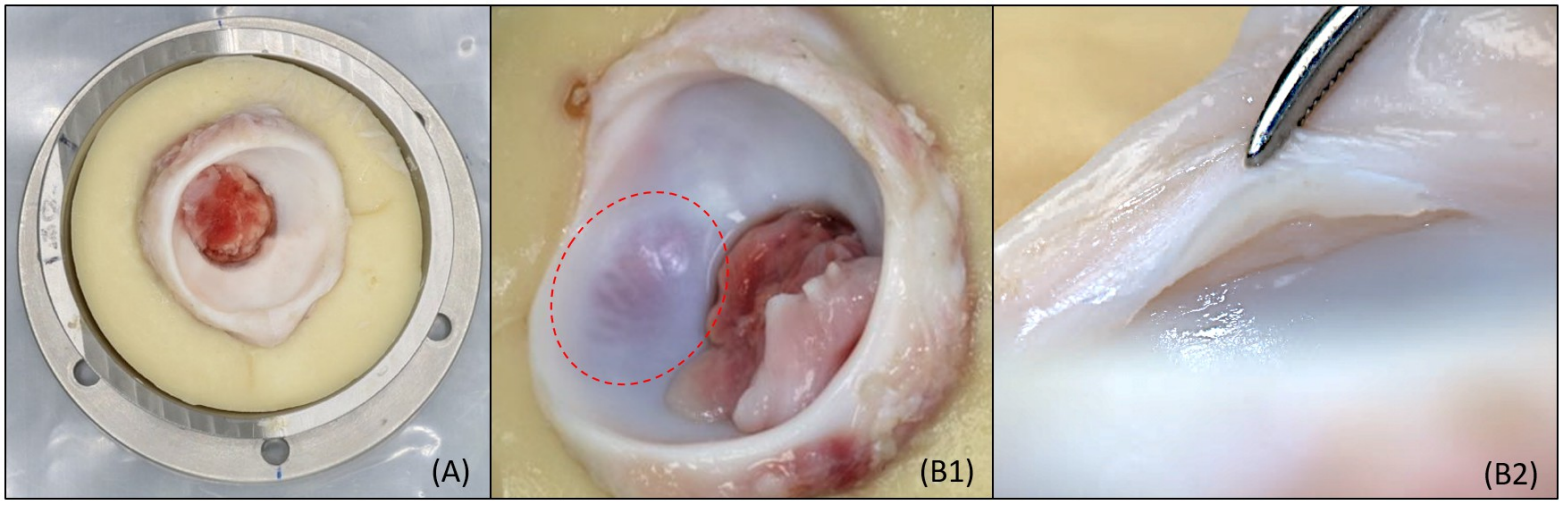
Sample photogrammetry of porcine acetabulum. (A) Wide lens photographs show the positioning of the sample and its overall condition prior to simulation. (B) Macro lens photograph shows the condition of the tissue in more detail. Images show examples of damage present after an OL+ simulation for 14,400 cycles. (B1) Cartilage blushing. (B2) Labral tear and separation of the chondrolabral junction.

Photogrammetry was used to characterise and catalogue the tissue condition and record the location/extent of damage labrum and/or articular cartilage pre, during (after 7,200 cycles), and post simulation (14,400 cycles) for all the samples. Multiple photographs were taken (Canon 750D DSLR with wide lens EF-S 28-70mm F3.5–5.6 and EF 100mm f 2.8 USM Macro) of both articular surfaces and the labrum following a standard protocol to ensure photos were captured in a consistent manner (Fig 9).

Output simulator data was monitored to ensure the input and output profiles were consistent. Input profiles, data generated in terms of input and output profiles and photographs for each test are openly available through the University of Leeds data repository [16].

## Results

### Method development

A methodology was developed that enabled the in vitro simulation of porcine hip with a standard walking cycle. It was possible to set up the simulation to ensure the COR of the femoral head and acetabular were consistently coincident with the COR of the simulator. During method development two technical failures (results excluded) occurred, one whereby there was cement ingress between the articular surfaces, and another where the femoral neck fractured.

### Effect of altered mechanical environment

**Fig 10:**
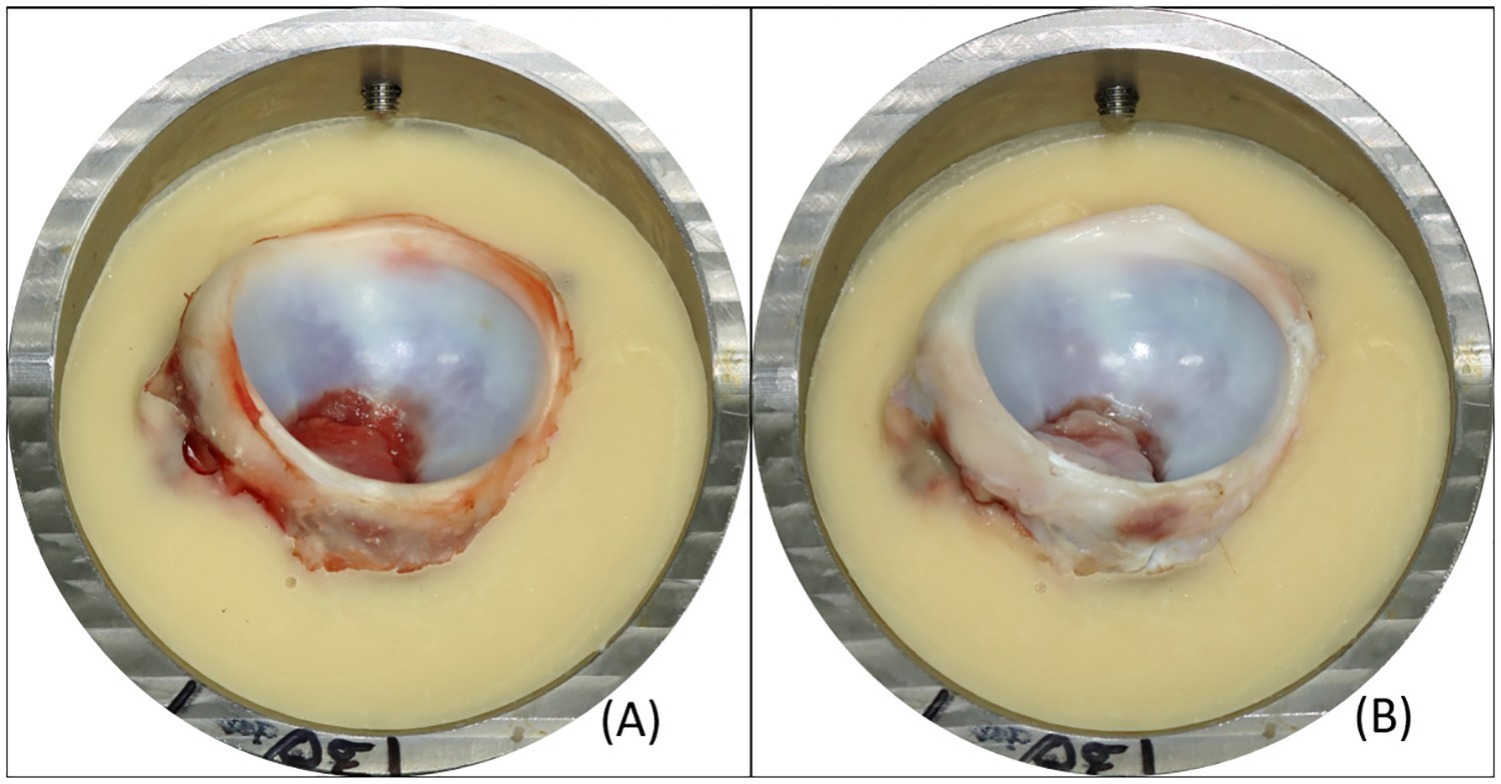
Photogrammetry of the acetabulum. Example images demonstrating (A) pre- and (B) post-test appearance of the samples for testing under normal (NOR) conditions.

Loading conditions were derived for “normal walking” (NOR) and two “overload” (OL and OL+) scenarios. Following testing under the normal (NOR) conditions (n = 4) for 14400 cycles there was no detectable damage to the articular surface or acetabular labrum. Representative images of the samples pre- and post- test are shown in Fig 10.

Following testing under the overload (OL) conditions, damage to the cartilage labral junction was observed. Tears and detachment of the labrum at the antero-superior region were observed (n = 4) at the end of the simulation (14400 cycles). However, no damage was detected when samples were reviewed at 7,200 cycles.

**Fig 11:**
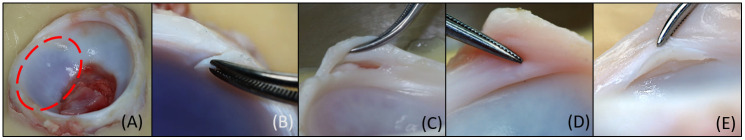
Types of acetabular damage observed following during simulations. (A) Cartilage blushing, (B) Labral tear, (C) Large labral tear, (D) Initial chondrolabral junction separation, (E) Large labral tear and chondrolabral junction separation.

**Fig 12:**
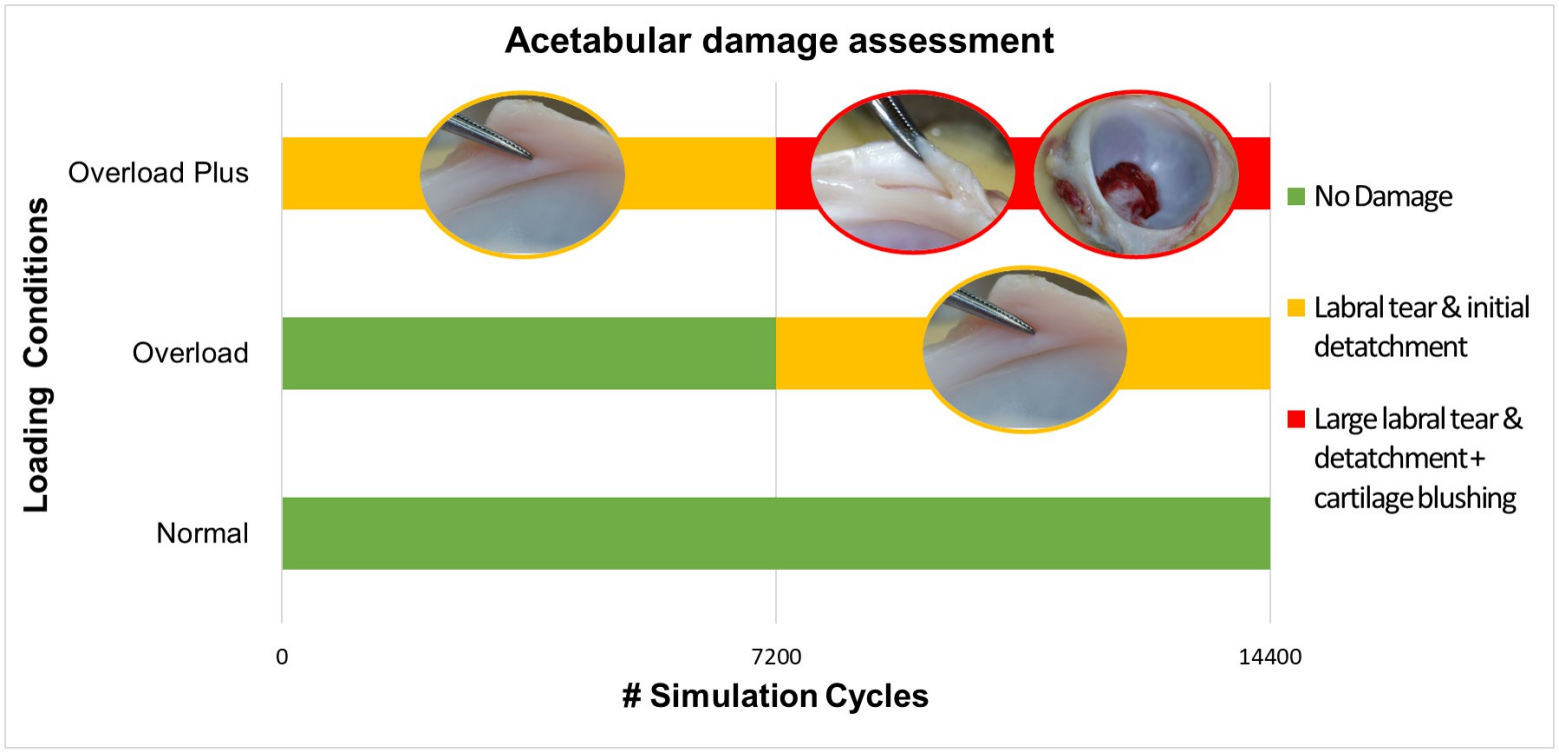
Acetabular damage assessment. Summary of the damage presented for the different loading scenarios.

In the case of all of the Overload Plus (OL+) scenarios, similar damage to that in the Overload (OL) conditions were observed, however, this was also larger in size and occurred earlier in the testing (observed at 7,2000 rather than 14,400 cycles), some signs of tearing were also observed after 2 hours of testing. Overload Plus conditions also showed cartilage bruising on the articular surface of the acetabulum. The type of damage to the articular surface and labral junction during the experiments at different time points are shown in Figs 11 and 12. All damage observed occurred in the anterior-superior region of the acetabulae regardless the loading condition.

## Discussion

Different hip pathologies can lead to a change in bony shape cause abnormal loading. This change in loading effects the tribology of the hip joint, and the increased stress distribution on the articular surface leads to mechanical failure and accelerates degeneration. Hip joint simulators have been widely used to perform tribology studies to evaluate the wear of total hip replacement components under different conditions, such as increased loading and RoM from daily activities and also the effects of changing the position of the THR components [6–8]. Such simulators have the capability to assess the effects of mechanical degradation in the natural hip. However, it is challenging to replicate the mechanics of the natural hip joint as it depends on the combination of numerous variables, such as individual sample variation, loading, RoM and sample size, among others. To date there have been few studies assessing the tribological behaviour of the natural hip in such experimental simulators [9, 17].

In the methodology presented in this study, a protocol was developed whereby the natural hip could be accurately and reproducibly set up in a simulator, accounting for sample variations. The bespoke fixtures ensured the COR was reliably set in all tests and was coincident with the COR of the simulator. The required disarticulation and the removal of the greater trochanter in sample preparation had the potential to compromise the stability of the joint, however, the method provided accurate positioning of the sample into the simulator and a very quick and reliable method for the macro evaluation of the articular surface (due to the disarticulation). The hip remained stable throughout testing.

Surface assessment and photogrammetry provided an accurate and fast approach to evaluate the cartilage and labral surfaces during and after simulation. The wide lens photographs provided a clear visualisation of the overall condition of the samples every time they were taken to identify the location of possible tissue damage. The macro photographs allowed observations of the detailed condition of a particular region of interest and to identify the type and severity of damage occurred. Photogrammetry took place in the same location as the experiment, allowing quick and minimal manipulation of the sample.

Following testing under the normal load condition, the porcine hip samples showed no evidence of damage. This provides evidence that this is an appropriate methodology and loading regime for in vitro studies on porcine hips and provides a benchmark to compare the effect of different loading conditions. The overload and overload plus conditions did cause increasing amounts of damage. This was is in agreement with previous in vitro testing of natural cartilage samples, that have demonstrated increasing damage with increasing load [14, 18, 19]. Most of the damage observed was located on the anterior-superior region of the acetabulae which is consistent with the location of damage reported clinically [20]. Damage observed between repeat tests conducted using the same conditions was consistent for all the samples, supporting this as a useful methodology to assess mechanical variables in the natural hip.

The developed methodology has some limitations. The removal of the great trochanter in the porcine samples was necessary to allow an appropriate RoM and avoid impingement between bones. This could affect the stability of the joint (however, this was not observed in these tests). The joint was disarticulated, which was required to fix the sample and to evaluate the articular surfaces through the test. It would have been ideal to maintain the capsule and teres ligament to preserve their function during the motion. The number of samples per loading condition was limited (n = 4), however development of the alignment and mounting methodologies used more samples (n = 10), therefore we were able to refine this process and reduce variability from joint position and ensure the damage observed was consequence of the loading and not of malpositioning of the sample. Finally, testing used cadaveric tissue and consideration to the differences this makes compared to living tissue should be considered. This may include the lubrication mechanism of the cartilage and recovery of the tissue from loading, and the contributions from viable cells in the cartilage. The altered lubrication will increase, stresses in the articular surfaces and we postulate damage occurs faster than in living tissue.

Future work will further assess the effects of altered loading and motion of the natural hip, the effects of misaligning the joint will also be assessed and can be used to gain insight into surgical processes used to reshape the hip (for example in the case of femoroacetabular impingement). The developed methods will be translated to human cadaveric tissue.
